# Prophage Diversity Across *Salmonella* and Verotoxin-Producing *Escherichia coli* in Agricultural Niches of British Columbia, Canada

**DOI:** 10.3389/fmicb.2022.853703

**Published:** 2022-07-22

**Authors:** Karen Fong, Yu Tong Lu, Thomas Brenner, Justin Falardeau, Siyun Wang

**Affiliations:** Food, Nutrition and Health, The University of British Columbia, Vancouver, BC, Canada

**Keywords:** prophage, phage, *Salmonella*, VTEC, CRISPR

## Abstract

Prophages have long been regarded as an important contributor to the evolution of *Salmonella* and Verotoxin-producing *E. coli* (VTEC), members of the *Enterobacteriaceae* that cause millions of cases of foodborne illness in North America. In *S*. Typhimurium, prophages provide many of the genes required for invasion; similarly, in VTEC, the Verotoxin-encoding genes are located in cryptic prophages. The ability of prophages to quickly acquire and lose genes have driven their rapid evolution, leading to highly diversified populations of phages that can infect distantly-related bacterial hosts. To defend against foreign genetic materials (i.e., phages), bacteria have evolved Clustered Regularly Interspaced Short Palindromic Repeats (CRISPR) immunity, consisting of variable spacer regions that match short nucleic acid sequences of invaders previously encountered. The number of spacer regions varies widely amongst *Enterobacteriaceae*, and there is currently no clear consensus if the accumulation of spacers is linked to genomic prophage abundance. Given the immense prophage diversity and contribution to bacterial host phenotypes, we analyzed the prophage sequences within 118 strains of *Salmonella* and VTEC, 117 of which are of agricultural origin. Overall, 130 unique prophage sequences were identified and they were found to be remarkably diverse with <50% nucleotide similarity, particularly with the Gifsy-1 group which was identified in several *Salmonella* serovars and interestingly, a strain of VTEC. Additionally, we identified a novel plasmid-like phage that carried antibiotic resistance and bacteriocin resistance genes. The strains analyzed carried at least six distinct spacers which did not possess homology to prophages identified in the same genome. In fact, only a fraction of all identified spacers (14%) possessed significant homology to known prophages. Regression models did not discern a correlation between spacer and prophage abundance in our strains, although the relatively high number of spacers in our strains (an average of 27 in *Salmonella* and 19 in VTEC) suggest that high rates of infection may occur in agricultural niches and be a contributing driver in bacterial evolution. Cumulatively, these results shed insight into prophage diversity of *Salmonella* and VTEC, which will have further implications when informing development of phage therapies against these foodborne pathogens.

## Introduction

Prophages, temperate phages that are integrated into a bacterial host genome, are recognized as one of the greatest drivers of bacterial diversity and evolution (Ramisetty and Sudhakari, 2019). Investigation of bacterial genomes have revealed that temperate phages are diverse, common, and abundant (Brueggemann et al., 2017; Crispim et al., 2018). For example, 53 complete prophages were identified in 47 analyzed genomes of *Desulfovibrio* (Crispim et al., 2018), and prophages were identified in all 482 strains of *Pneumococci* studied (Brueggemann et al., 2017). Under various experimental environments, prophages also afforded their bacterial host with a range of fitness benefits, such as integration of antibiotic resistance genes encoding resistance to kanamycin, chloramphenicol and ampicillin (Wendling et al., 2020). Excision and release of free prophages that then lysed competing bacteria was also advantageous from a population standpoint (Wendling et al., 2020). Interestingly, prophage carriage often impedes superinfection from other phages; a phenomenon which provides a great survival benefit in the event the invading phage is massively virulent (Ramisetty and Sudhakari, 2019). Indeed, prophages possess novel genetic materials which have contributed greatly to host phenotype diversity. For example, the Verotoxin-encoding genes of Verotoxin-producing *Escherichia coli* (VTEC) are located in lambdoid prophages and the production of these toxins occur following prophage excision from the bacterial genome (Łos et al., 2013). Strains of *Salmonella enterica* serovar Typhimurium also contain an array of prophages that are critical for host virulence and fitness, such as the Gifsy phages (Hiley et al., 2014).

Non-typhoidal *Salmonella* and VTEC are foodborne pathogens which cause significant burdens on both the Canadian health care system and economy, ranking third and fourth, respectively, for the number of deaths attributed to foodborne illness (Government of Canada, 2016). In recent years, antibiotic resistance has risen to dangerously high levels and deaths from common infections are increasingly attributed to antibiotic resistance (World Health Organization, 2018). The growing inadequacy of antibiotics has paved the way for novel therapies that rely on antibiotic alternatives. Many studies have focused on application of lytic phages for direct killing of their bacterial hosts, however, host resistance still occurs (Fong et al., 2017, 2020).

Prophages may also be employed as antimicrobials (Hu et al., 2021). When environmental triggers inflict stress upon the host cell (e.g., UV light, antibiotic treatment) and induce the bacterial SOS response, prophage induction occurs, where the prophage excises from the genome, ultimately leads to host cell lysis and death (Fu et al., 2019). Somewhat paradoxically, prophage induction plays a critical role in the virulence of VTEC as its pathogenicity requires prophage release from the bacterial cell, which simultaneously releases the prophage-encoded Verotoxin (Łos et al., 2013). Because the host cell is killed upon prophage induction, strategies to induce prophage release have been explored as a means of biocontrol. In a study evaluating the effect of stresses on the production of prophage particles, inducing agents including heat, hydrogen chloride, lactic acid, hydrogen peroxide and high hydrostatic pressure reduced VTEC O104:H4 by 1 to 2 log CFU/ml by release of prophage (Fang et al., 2017). More recently, a proof-of-concept study by Cadieux et al. (2018) found that mitomycin C was the strongest inducing agent of VTEC and *Salmonella* prophages at a minimum concentration of 0.5 ug/mL. This resulted in a 1.5 and 3 log CFU/g reduction of *Salmonella* and VTEC on tomatoes, respectively. Similar reductions of 1 and 2 log CFU/g for VTEC and *Salmonella*, respectively, were also observed on spinach.

Clustered regularly interspaced short palindromic repeats (CRISPR) are repeat sequences that provide adaptive immunity against invasive foreign elements such as phages (Fu et al., 2017). The CRISPR-Cas system comprises two separate components: Cas-proteins that directly cleave foreign nucleic acid; and the CRISPR array that is an invader recognition tool (Touchon et al., 2011). Because CRISPRs are widespread in ~40% of bacteria (Touchon and Rocha, 2010), we probed the number and type of CRISPR arrays in our subset of bacterial strains in an effort to discern an association between lysogeny and carriage of CRISPRs. The CRISPR array affords the bacterial cell with “memory” for defense against foreign nucleic acid (e.g., plasmids and phages) by incorporating unique protospacers of invaders previously encountered (Touchon et al., 2011). Of particular interest are the spacer regions, as it is known these regions are usually identical to sequences possessed by mobile genetic elements (Louwen et al., 2014).

Given the survival advantages of prophage carriage and the advent of such novel approaches to phage-based biocontrol, it is important to gain a deeper understanding of the prophages of both VTEC and *Salmonella*. Previous work has highlighted the orthologous prophages of *Escherichia* and *Salmonella* (Bobay et al., 2014), and these genera include pathogens with highly plastic genomes (Touchon and Rocha, 2010). The enhanced availability of sequenced bacterial and phage genomes will undoubtedly offer additional insights into phage immunity. Here, we probe the prophage-related genetic elements of 118 strains (117 of agricultural origin) of VTEC and *Salmonella* in an effort to understand the abundance and diversity of the prophage repertoire in these bacterial pathogens. We also identify host CRISPR-Cas elements to discern an association between CRISPR arrays and the incidence of lysogeny.

## Materials and Methods

### Bacterial Strains

Bacterial strains used in this study are listed in Table 1. *Salmonella* isolates (n=50) were previously isolated and analyzed from poultry and environmental samples by Brenner et al. (2020). Strains were paired-end sequenced on an Illumina NextSeq with a coverage of 15X. Reads were assembled with SPAdes v.3.10 and draft sequences assemblies annotated with the NCBI Prokaryotic Genome Annotation Pipeline (Bankevich et al., 2012). *Salmonella* sequences were deposited into Genbank under BioProject PRJNA224116.

**Table 1:** *Salmonella* and VTEC strains used in this study.

***Salmonella***		**VTEC**	
**Strain**	**Serotype**	**Strain**	**Serotype**
S01^a^	Brandenburg	EDL933	O157:H7
S02^a^	I:4,5,12:i:-	SN002^c^	O177:NM
S03^a^	Reading	SN007^c^	O157:NM
S04^a^	Reading	SN017^c^	O8:H19
S05^a^	Typhimurium	SN021^c^	O168:H8
S06^a^	Typhimurium	SN061^c^	O26:H11
S07^a^	Rissen	SN062^c^	O103:H11
S08^a^	Kentucky	SN073^c^	O116:H25
S09^a^	Kentucky	SN127^c^	O5:NM
S10^a^	Kentucky	SN130^c^	O111:NM
S11^a^	Kentucky	SN141^c^	O111:NM
S12^a^	Kentucky	SN142^c^	O111:H8
S13^a^	Kentucky	SN149^c^	O98:NM
S14^a^	Kentucky	SN158^c^	O5:NM
S15^a^	Kentucky	SN173^c^	O130:H8
S16^a^	Kentucky	SN174^c^	O22:H8
S17^a^	Kentucky	SN182^c^	O174:H21
S18^a^	Kentucky	SN200^c^	O163:NM
S19^a^	Kentucky	SN203^c^	O163:NM
S20^a^	Kentucky	SN204^c^	OR:NM
S21^a^	Kentucky	SN218^c^	OR:NM
S22^a^	Kentucky	SN220^c^	O163:H19
S23^a^	Kentucky	SN230^c^	O69:H11
S24^a^	Kentucky	SN231^c^	OR:NM
S25^a^	Kentucky	SN232^c^	OR:NM
S26^a^	Enteritidis	SN235^c^	OR:NM
S27^a^	Enteritidis	SN245^c^	OR:H21
S28^a^	Enteritidis	SN258^c^	O5:NM
S29^a^	Enteritidis	SN265^c^	O8:H19
S30^a^	Enteritidis	SN300^c^	O128:H2
S31^a^	Enteritidis	SN305^c^	O26:H11
S32^a^	Enteritidis	SN306^c^	O26:H11
S33^a^	Enteritidis	SN321^c^	O151:H12
S34^a^	Enteritidis	SN354^c^	O98:NM
S35^a^	Enteritidis	SN408^c^	O98:NM
S36^a^	Enteritidis	SN412^c^	O157:H7
S37^a^	Enteritidis	SN440^c^	O163:H19
S38^a^	Enteritidis	SN443^c^	O165:H25
S39^a^	Enteritidis	SN465^c^	O165:NM
S40^a^	Enteritidis	SN496^c^	O157:H7
S41^a^	Enteritidis	SN534^c^	O103:H2
S42^a^	Enteritidis	SN539^c^	O103:H25
S43^a^	Enteritidis	SN545^c^	O26:H11
S44^a^	Enteritidis	SN550^c^	O165:NM
cS45^a^	Enteritidis	SN556^c^	O174:H8
S46^a^	Enteritidis	SN570^c^	O163:H19
S47^a^	Enteritidis	SN573^c^	O128:H2
S48^a^	Enteritidis	SN576^c^	O111:NM
S49^a^	Enteritidis	SN583^c^	O8:H9
S50^a^	Enteritidis	SN586^c^	O103:H25
		SN598^c^	O103:H2
		SN601^c^	O103:H2
		SN608^c^	O103:H2
		V-JF-003^d^	O116:H25
		V-JF-005^d^	O103:H2
		V-JF-007^d^	O103:H2
		V-JF-008^d^	O103:H2
		V-JF-010^d^	O109:H5
		V-JF-012^d^	O116:H25
		V-JF-017^d^	O76:H19
		V-JF-021^d^	O69:H11
		V-JF-025^d^	O69:H11
		V-JF-029^d^	O69:H11
		V-JF-033^d^	O34:H32
		V-JF-036^d^	O34:H32
		V-JF-039^d^	O22:H8
		V-JF-043^d^	O153:NM
		V-JF-047^d^	O153:NM

*^a^Strains obtained from Brenner et al. (2020). Bioproject ID: PRJNA224116*;

*^b^Clinical origin*;

*^c^Strains obtained from Nadya et al. (2016). Bioproject ID: PRJNA287560*;

*^d^ Strains obtained from Falardeau et al. (2017). Bioproject ID: PRJNA649237*.

The VTEC strains were isolated and analyzed previously in agricultural environments (irrigation water, sediment) by Nadya et al. (2016) and Falardeau et al. (2017) (Table 1). One clinical isolate from a previous foodborne outbreak, *E. coli* O157:H7 EDL933, was used as a reference strain because it was the first VTEC strain to be sequenced and has since been studied across many laboratories worldwide (Perna et al., 2001). VTEC strains beginning with “V-JF” were paired-end sequenced on an Illumina HiSeq with a coverage of 200X. Reads were assembled with SPAdes v.3.10 and draft sequence assemblies annotated with the NCBI Prokaryotic Genome Annotation Pipeline (PGAP). VTEC strains beginning with “SN” were subjected to paired-end sequencing on an Illumina MiSeq and assembled de novo with Spades v.3.1 (Bankevich et al., 2012). Draft sequence assemblies were annotated with Prokka v.1.10 (Seemann, 2014). VTEC sequences were deposited into Genbank under BioProject numbers PRJNA649237 and PRJNA287560.

### Bioinformatics Analysis

PHASTER (PHAge Search Tool Enhanced Release) was used to identify intact prophages in the assembled draft genomes (Arndt et al., 2016). We used stringent criteria for prophage identification as described by Colavecchio et al. (2017b). Only prophages designated “intact” were used for further analysis. Hits to known phages were identified through PHASTER and subsequently confirmed with NCBI BLAST (Altschul et al., 1990).

All identified prophages were grouped if they were identified by BLAST best hit. If phages were identified by BLAST best hit and possessed 95–99.9% similarity over 50% query, they were designated variants and assigned a Roman numeral: I, II, III and so on. Nucleotide similarity was determined using the Clustal Omega alignment tool (Madeira et al., 2022), freely available at https://www.ebi.ac.uk/Tools/msa/clustalo/. Genes were annotated automatically with RAST (https://rast.nmpdr.org/) (Aziz et al., 2008).

### Phylogenetic Analysis

The phylogenetic tree was constructed in R using the Ape package (Paradis and Schliep, 2019). Sequences were aligned using the ClustalW algorithm and the phylogenetic tree constructed using the Maximum-Likelihood method, employing 1,000 bootstrap replicates. The tree was imported into FigTree for additional rendering (Rambaut, 2007). Clusters were identified with ClusterPicker (Ragonnet-Cronin et al., 2013) using an inter-cluster threshold of 50% nucleotide identity (Fong et al., 2019). Comparative gene maps were constructed in R with the GeneplotR package (Guy et al., 2010) and Geneious Prime v.2022.0.2. Circular genomic visualizations and dotplot alignments were constructed with Geneious Prime v.2022.0.2 (www.geneious.com).

### CRISPR Analysis

Bacterial CRISPR-Cas arrays were identified with CRISPRFinder (Grissa et al., 2007) using default parameters. Spacer sequences were assessed for homology to known phages using NCBI BLAST. The number of spacer sequences of VTEC and *Salmonella* were statistically compared with the Student's *t*-test in JMP version 11.1.1 (SAS Institute, Inc., Cary, NC, United States). A *P*-value of ≤0.05 was considered statistically significant.

## Results and Discussion

### General Prophage Characterization

Cumulatively, a total of 289 prophages were identified with bioinformatics analysis in our collection of *Salmonella* (*n* = 50) and VTEC (*n* = 68) strains (Supplementary Tables S1, S2). These were grouped into 130 unique (95% nucleotide identity over 50% query cover or lower) prophage sequences. Similar prophages within the same variant group were identified in diverse hosts (Supplementary Table S1) which may signify multiple phage/host interaction implications such as similar host ranges, common phage ancestry and recent or stable horizontal exchange and host acquisition (Hendrix et al., 1999). It is important to emphasize that phages were not induced experimentally, nevertheless, the intact prophages identified in this study would theoretically be capable of excision. As with all *in silico* prophage prediction tools, potential underrepresentation and misinterpretation of prophage diversity and abundance exists (i.e., split between two contigs in draft assemblies) and has been discussed extensively by Hurwitz et al. (2018). The prophages uncovered in this work provide a preliminary understanding of relationships in their respective hosts.

**Figure 1:**
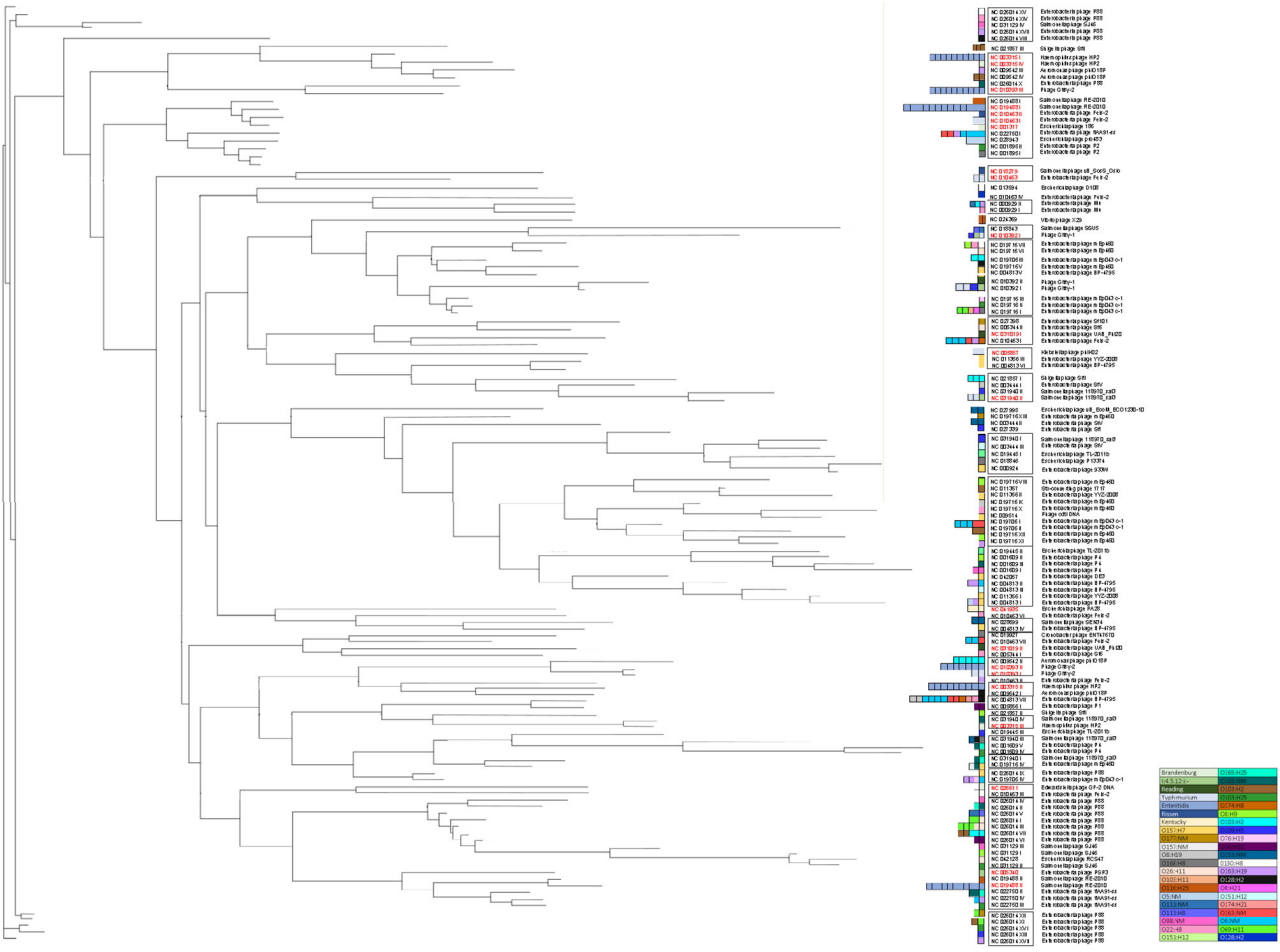
Maximum-likelihood tree of 130 prophages identified in this study. For clarity, bootstrap values (all >70%) are not shown. Scale bar represents the average number of nucleotide substitutions per site. Red font indicates prophages identified in our Salmonella strains. Colored boxes next to taxa names indicate serotype abundance and in which the prophages were identified.

Prophages were clustered into 26 clades on the basis of 50% nucleotide similarity with 13 genomic singletons, highlighting the diversity in our subset of strains (Figure 1). Interestingly, prophages clustered together regardless of the bacterial genus, indicating that *Salmonella* and VTEC share genetically similar prophages with broad host ranges capable of inter-genus infectivity. Furthermore, these data suggest active dissemination of prophages amongst genetically distinct bacterial populations that may cohabitate similar niches, such as agricultural sites. We found prophages in *Salmonella* and VTEC that were most closely related to those of other hosts, such as *Klebsiella, Haemophilus* and *Vibrio*. The identification of genetically similar prophages amongst distinct bacteria may broaden the host range when designing future prophage therapies (e.g., engineering broad-range prophage induction agents) against foodborne pathogens (Hu et al., 2021). Furthermore, the discovery of distant hosts with closely-associated clinical outcomes may be attributed to the impact of the host environment in phage-host interactions.

We detected 13 unique prophages in 50 genomes of *Salmonella* (Supplementary Table S1). The abundance and type of prophage correlated with *Salmonella* serotype (i.e., prophage typing) (Figure 1, Thapa and Mohammed, 2019). Some prophages (NC 003315 I, NC 003315 II, NC 010393 II, NC 010393 III, NC 019488 I, NC 019488 II and NC 009542 II) were exclusively detected in strains of the same serotype (Figure 1). Polylysogeny (i.e., the carriage of more than one prophage) was also common; on average, 1.94 prophages were detected per *Salmonella* genome, which is lower than what has been reported (Bobay et al., 2013; Mottawea et al., 2018). Extremely divergent phages were also identified within the same genome; strain S01 contained five intact phages with an average nucleotide similarity of 51.5%. Similarly, S05 also contained five phages with an average similarity of 37.5%. These data indicate that phages with novel and diverse genetic materials are present in the genomes of our *Salmonella* collection. Compared to VTEC, we detected far fewer unique prophages in *Salmonella*. This may be due to several factors. For instance, *E. coli* may contain more preferable genomic integration sites and/or compatible genomic content (e.g., tRNAs, similar GC content) which has been demonstrated to favor phage infection and integration (Cardinale and Duffy, 2011). As part of the host immune response, superinfection exclusion systems also restrict superinfection by similar phages (Seed, 2015).

VTEC strain EDL933 was used in our prophage analyses as it is a well-characterized strain possessing genetically diverse prophages (Saile et al., 2016). Overall, 66 of 68 VTEC strains possessed at least one prophage. We did not identify any prophages in SN496 nor SN573, indicating these prophages may not be intact and/or defective. In a screening of 40 VTEC isolates by Zhang et al. (2020), 15.3 and 9.3% of prophages were incomplete and questionable, respectively. It currently remains unclear whether these prophages are inducible, however, Asadulghani et al. (2009) induced several defective prophages of VTEC O157 and found these phages maintained their virulence after the induction.

Clinical type strain EDL933 possessed 11 prophages, the most prophages of all strains tested. As the majority of our VTEC strains were isolated predominantly from the agricultural environment, these results suggest that these prophages may confer advantages to the host such that it allows for enhanced persistence in such environments.

### Variants of Gifsy-Like Phages

**Figure 2:**
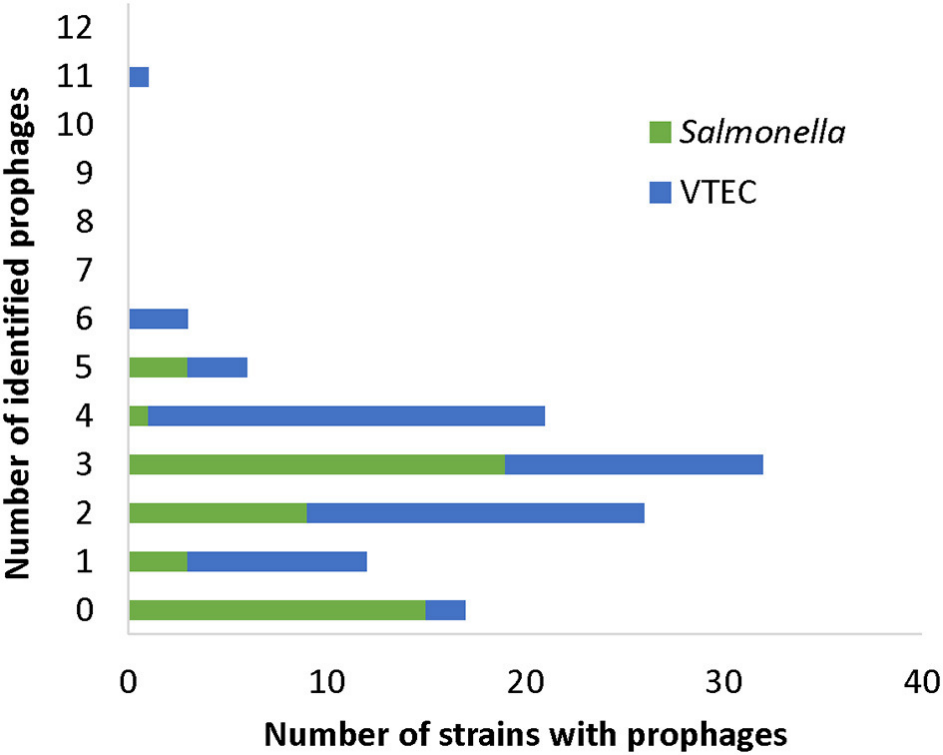
Frequency histogram of strains carrying prophages.

**Figure 3:**
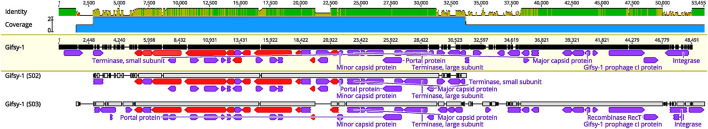
Comparisons between Gifsy-1 and related variants in S02 and S03. ORFs in red represent tail protein regions in the respective phages. Structural proteins and lysogeny-related genes are indicated.

On average, the incidence of prophage carriage in *Salmonella* was 1.94 per genome and 3.03 per VTEC genome (Figure 2). Prophage profiles clustered closely with serotype (i.e., number and type of phage correlated with bacterial serotype) (Figures 1, 2 and Supplementary Table S1), however, some prophages and their variants (e.g., NC_010392; Gifsy-1 and NC_010393; Gifsy-2) were not serotype-dependent and were identified in several strains representing different serotypes. Gifsy-1 and−2 -like phages are best known for contributing to the virulence of *S*. Typhimurium (Ho and Slauch, 2001), however in the present study they were identified in the genomes of *S*. Reading, *S*. Typhimurium, *S*. Enteritidis and VTEC. The variants differed greatly, with NC_010393 (I) possessing a genome size of ~16 kb, almost 50% smaller than variants (II) and (III). Further analysis revealed that these genes in variant (I) were mostly structural, while variants (II) and (III) also carried a slew of accessory genes for host invasion (e.g., *sopE*). Given that the annotation assigned mostly structural gene predictions, the carriage of variant (I) does not indicate the presence of any obvious host survival advantages but may provide an indirect mutualistic benefit if lysogeny is stably maintained. Diversity in Gifsy-1 was also greatly apparent (Figure 3) with variant (I) ~30% shorter than variant (II). An intact lysogeny cassette was not identified in this variant, although major structural and host recognition genes were identified (e.g., minor and major capsid proteins, portal proteins, tail fibers). Gifsy-1 was not only found in our subset of *Salmonella* strains but also in closely related to Gifsy-1 prophage in VTEC strain V-JF-010, which, to the best of our knowledge, has not been documented in *E. coli* before. Previous work has revealed that *E. coli* requires *Salmonella* OmpC protein for Gifsy prophage to bind (Ho and Slauch, 2001), however, upon homology analysis with NCBI BLAST no homologs were identified in strain V-JF-010. Further work is needed to elucidate the receptor of interest, and also to identify putative receptor sites in other bacterial genera, particularly in type phages such as Gifsy-like viruses that may confer virulence. The identification of such sites in taxonomically distinct species of bacteria is important for the design of prophage-engineered biocontrol strategies, an area of study still currently in its infancy (Cadieux et al., 2018).

### Carriage of Host-Derived Virulence Factors

Various genetic elements encoding an array of functions (i.e., host virulence, antimicrobial resistance) were identified in prophages of both *Salmonella* and VTEC (Table 2). Carriage of these virulence factors is problematic as these genes may be readily transduced throughout a bacterial population, potentially impacting host fitness such that it allows for greater survival in a variety of conditions. Phages may carry an array of genes that confer a host advantage (Colavecchio et al., 2017a; Gómez-Gómez et al., 2019; Wendling et al., 2020). For instance, prophages highly related to Gifsy-1, Gifsy-2 and Gifsy-3 are conserved within *S*. Typhimurium and contribute significantly to host virulence (Hiley et al., 2014). A previous study by Ross and Topp (2015) also observed the abundance of antibiotic-resistance elements in phages sourced from soil, suggesting that the agricultural environment may be a potent source of such genes.

**Table 2:** Putative virulence genes carried by the prophages identified in this study.

**Phage**	**Variant**	**Strain(s)**	**Annotation**
NC_010392	I	S02, S05, S06	Putative virulence factor
NC_011356	I	EDL933	Putative secreted effector protein
NC_011356	I	EDL933	Putative secreted effector protein
NC_000924	I	EDL933	Bor protein
NC_042057	I	EDL933	Bor protein
NC_042057	II	EDL933	Small multidrug resistance (SMR) efflux transporter = > EmrE, broad substrate specificity
NC_027339	I	V-JF-010	Shiga toxin subunit A
NC_027339	I	V-JF-010	Shiga toxin subunit B
NC_019716	V	SN300	Shiga toxin subunit A
NC_019716	V	SN300	Shiga toxin subunit B
NC_019716	XII	SN583	Shiga toxin subunit A
NC_019716	XII	SN583	Shiga toxin subunit B
NC_019716	XI	SN570	Shiga toxin subunit A
NC_019716	XI	SN570	Shiga toxin subunit B
NC_019716	VI	SN245	Shiga toxin subunit A
NC_019716	VI	SN245	Shiga toxin subunit B
NC_019716	VII	SN583, SN173, SN174	Shiga toxin subunit A
NC_019716	VII	SN583, SN173, SN174	Shiga toxin subunit B
NC_018846	I	SN021	Shiga toxin subunit A
NC_018846	I	SN021	Shiga toxin subunit B
NC_005856		V-JF-033, V-JF-036	Per-activated serine protease autotransporter enterotoxin EspC / autotransporter domain, T5aSS type secretion
NC_004813	VI	EDL933	Shiga toxin subunit A
NC_004813	VI	EDL933	Shiga toxin subunit B
NC_031129	II	SN539	Putative tellurite/colicin resistance
NC_018843	I	SN141, SN142	Putative tellurite/colicin resistance

**Figure 4:**
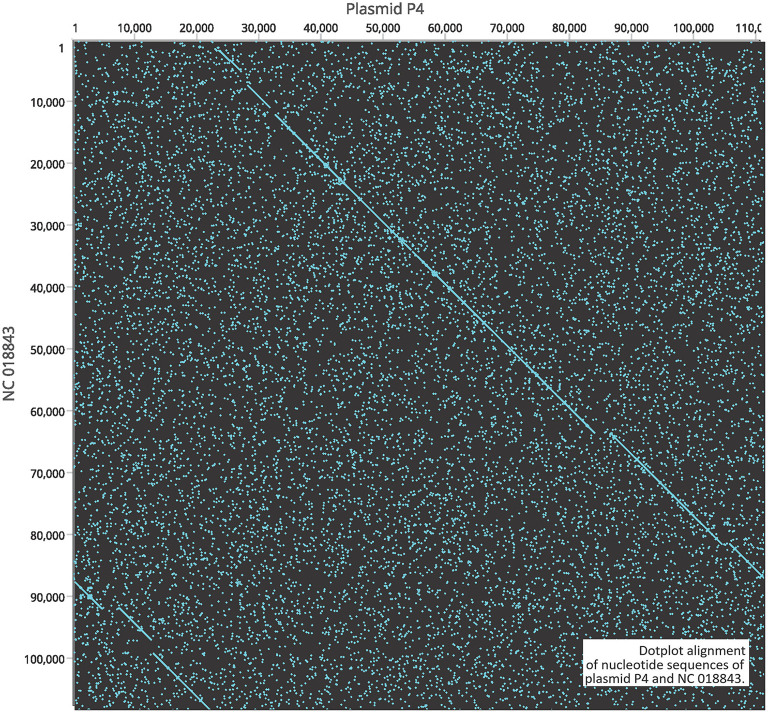
Dotplot alignment of nucleotide sequences of plasmid P4 and NC 018843.

**Figure 5:**
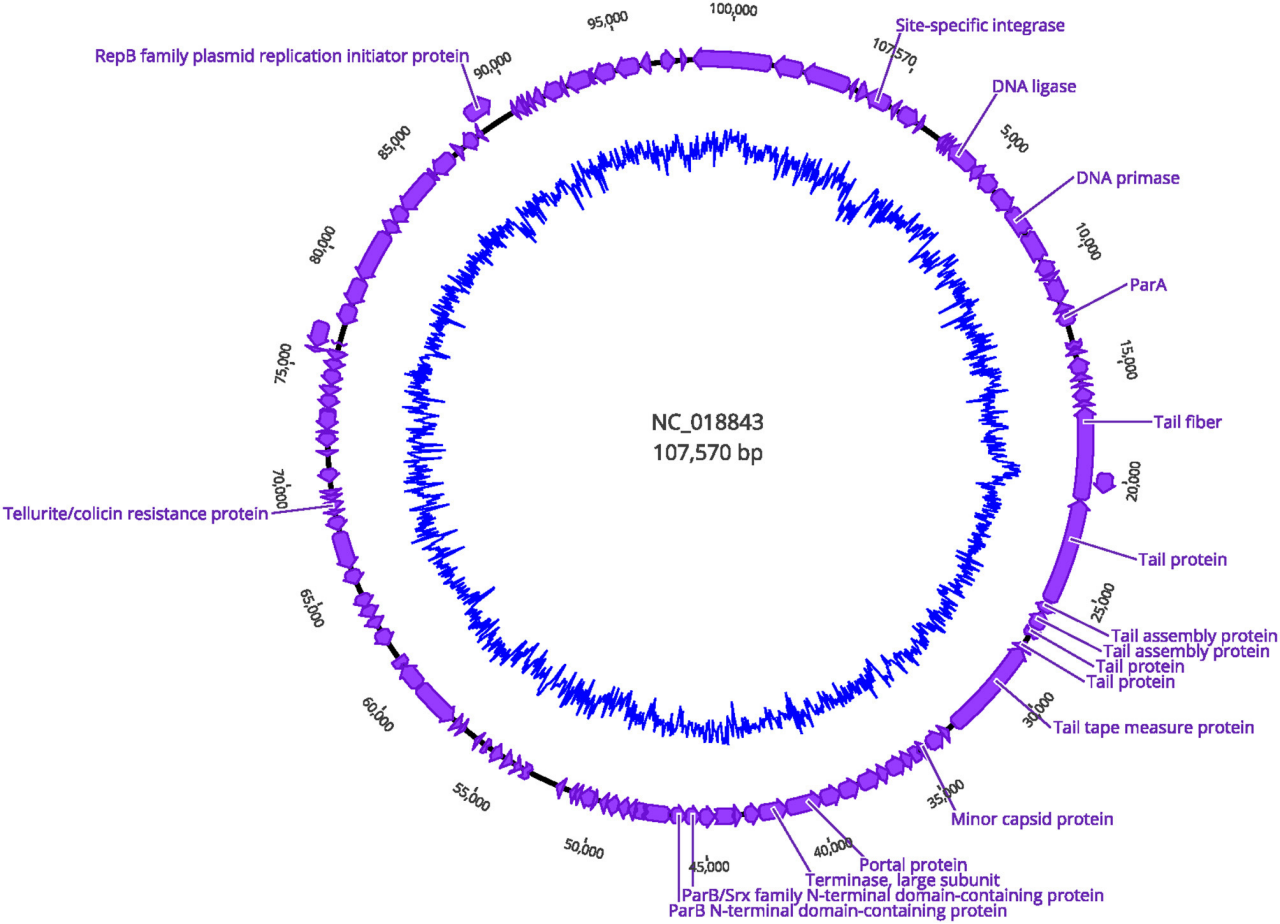
Circular genome visualization of prophage SSU5 identified in SN141 and SN142. Structural genes and genes encoding host genome integration are indicated. Inner circle represents GC content.

A gene encoding putative tellurite and colicin resistance was functionally annotated in prophage SSU5 of SN141 and SN142 (Table 2). Interestingly, we also identified partitioning genes *parA* and *parB*. A high degree of homology to plasmid p4 was observed with demonstrated genomic rearrangements relative to each other (Figure 4). Plasmid p4 was originally isolated from an extended spectrum β-lactamase-producing strain of *E. coli* (Brolund et al., 2019; Accession: CP023851.1). Previous studies have observed the high degree of genomic similarity between SSU5 and plasmid pHCM2 (Kidgell et al., 2002; Kim et al., 2012), however, this variant of SSU5 appears to be most closely related to plasmid p4. SSU5 is 107,570 bp in length, with 130 open reading frames (Figure 5). Its putative roles as both a plasmid and phage may represent more avenues for transmission of genes that confer increased host fitness and warrants further investigation.

Tellurite resistance compounds have been found in several genera of bacteria, including *E. coli, Salmonella, Shigella* and *Vibrio*. Though uncommon, these compounds have been found in the chromosome or on plasmids (Chasteen et al., 2009). Experimentally, tellurite is used as a selective agent for the isolation of many pathogens, including *E. coli* O157:H7 (Turkovicova et al., 2016). Compared to the wild type strain, *E. coli* BL21 possessing the *ter* operon (*terZABCDEF*; responsible for conferring tellurite resistance) was able to grow in the presence of extremely high concentrations of tellurite (minimum inhibitory concentration of ~4 mM) (Turkovicova et al., 2016). In the environment, tellurium is relatively rare, however, its soluble salts such as potassium tellurite were used clinically as an antimicrobial agent in the past (Valkova et al., 2007). Additionally, it was shown previously that *ter* genes enhanced the ability of *E. coli* to survive in macrophages (Valkova et al., 2007).

It is interesting to note that the *ter* operon is functionally diverse and may encode for a variety of other bacterial phenotypes. The *ter* genes play a coordinated role in stress resistance and may offer resistance against a broad spectrum of agents, including colicins (small antimicrobial peptides (i.e., bacteriocins) produced by *E. coli* to kill non-host *E. coli* cells) (Jin et al., 2018) and phages by mounting a restriction or suicidal action upon phage infection (Anantharaman et al., 2012). Given the increasing demand for antibiotic alternatives in sectors such as food processing and clinical medicine, the dissemination of phage resistance genes in bacterial hosts can be problematic as it may limit the efficacy of phage-based treatments for host elimination (e.g., direct phage application). Because SSU5 may be able to act intracellularly as a plasmid and a phage, multiple avenues for dissemination throughout a bacterial population exist and may therefore amplify the spread of critically important genes such as those encoding antimicrobial resistance. Such phages may help to drive evolution of bacterial populations in certain environments.

### Spacer Elements

**Figure 6:**
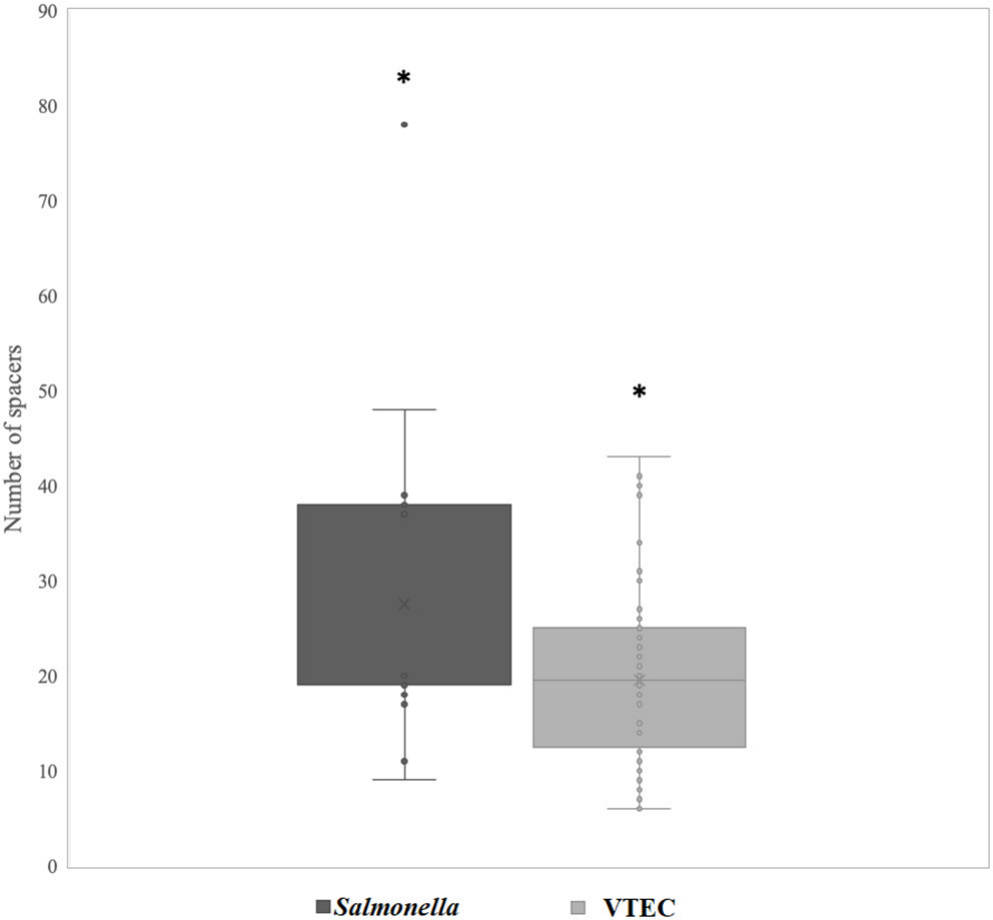
Number of spacer regions in Salmonella and VTEC. Asterisks indicate significance below α = 0.05 (Student's *t*-test).

The CRISPR loci is exploited for various analyses, such as microbial typing and tracking (Dion et al., 2021). Since spacer regions are hypervariable and provides historical information on phage resistance, we screened the spacer regions of identified CRISPR arrays with the aim to elucidate and characterize possible associations between spacer elements, prophage lysogeny and strain-specific differences in our dataset. All strains contained at least six unique spacers (Figure 6 and Supplementary Table S3). *Salmonella* contained significantly more spacers (an average of 27 regions) than VTEC (an average of 19 regions) (p <0.05). Previous work has revealed that in over >600 strains of *Salmonella* representing four serotypes, an average of 16 Class 1 and 20 Class 2 systems were identified (Shariat et al., 2015). More recent work also elucidated the diversity of spacers among various serotypes of *Salmonella* and identified a high number of spacers (440 and 330 unique spacers within 2221 and 2211 of CRISPR 1 and CRISPR2 arrays, respectively) in all *Salmonella* strains analyzed (Kushwaha et al., 2020). Many of the core CRISPR-associated (Cas) proteins that comprise the effector complex have evolved in line with the arms race against evolving phages (Koonin and Makarova, 2019). Thus, on the basis of Cas proteins and array organization, CRISPR defense systems have been categorized into two groups, Class 1 and Class 2 that are respectively subdivided into types I, III, IV and types II, V, VI (Makarova and Koonin, 2015). In a separate report surveying 100 strains of *E. coli*, 745 unique spacers were identified (Díez-Villaseñor et al., 2010). This is in stark contrast to a previous study by Touchon and Rocha (2010), where they observed no more than three CRISPRs in 51 genomes of *Salmonella* and *Escherichia*. These conflicting data may be because of several factors, not the least being differences in serotype, pathogenicity, and source of isolation of the strains tested. Zeng et al. (2017) observed that compared to clinical isolates (*n* = 17 spacers), food isolates of *Cronobacter sakazakii* possessed a significantly greater number of spacers in their CRISPR loci (*n* = 30). Correspondingly, foodborne isolates possessed less prophages (*n* = 2.81) compared to clinical isolates (*n* = 4.15). Enhanced evolution in CRISPR loci is important to consider when developing strategies such as phage cocktails for pathogen control because a key criterion for successful biocontrol is that resistance to phages is absent or delayed (Fong et al., 2019). It should be noted, however, that our bacterial strains of agricultural origin are draft genomes and may potentially underrepresent the true number of spacer regions. Nevertheless, our findings provide a preliminary basis for further probing of such genomes.

**Figure 7:**
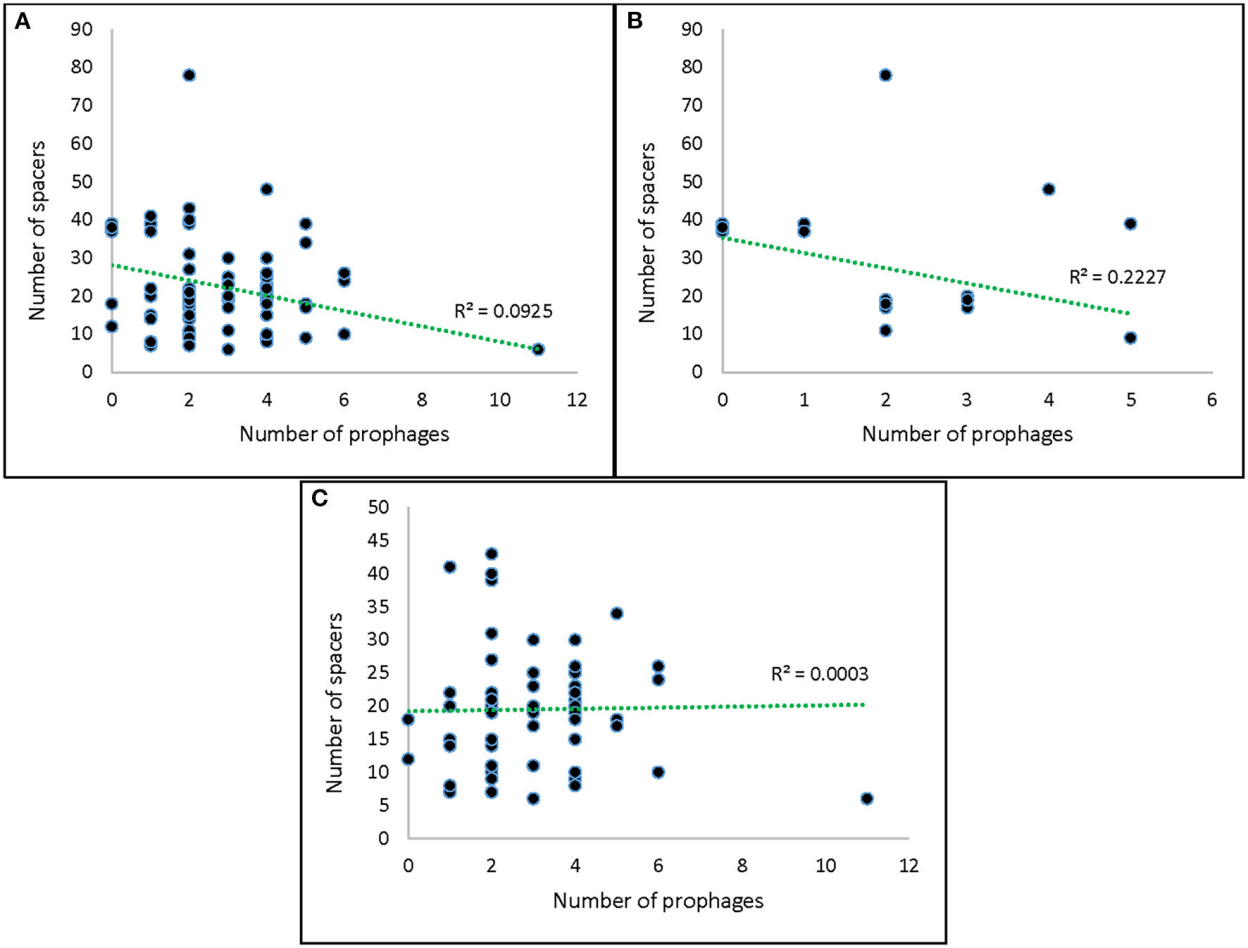
Correlation between number of spacers and number of prophages. **(A)** All strains; **(B)** Salmonella strains; **(C)** VTEC strains.

Spacers with significant homology to the identified prophages in this study were not observed, and we did not identify any self-targeting spacers. Surprisingly, only a small subset of strains (14%) encoded spacers with homology to known prophages, and these were only identified in VTEC (Supplementary Table S3). It should be noted that the scarcity of sequenced prophages in public databases may have supported this observation (Zeng et al., 2017). We also did not observe a significant correlation between spacer and prophage abundance in our strains (Figure 7). *In silico* work by Wang et al. (2020) also did not discern an association between these two elements in *Bifidobacterium pseudocatenulatum*. Despite their high degree of conservation, the role of CRISPR-Cas systems in prophage immunity has been questioned previously (Shariat et al., 2015), which has led to the hypothesis that these systems in *Salmonella* and VTEC may have alternative functions yet to be clarified (Touchon and Rocha, 2010; Shariat et al., 2015).

## Conclusion

We have described here the identification and analysis of 130 unique prophages found in the genomes of 118 strains of *Salmonella* and VTEC, 117 of which are of agricultural origin. These prophages are diverse, with many prophages sharing a common host between *Salmonella*, VTEC and other bacteria, including *Vibrio, Shigella* and *Klebsiella*. The prophages identified contain novel attributes and genes that may have far-reaching impacts on host phenotype. Further probing of the host CRISPR elements revealed an abundance of spacers in our strains, which provides preliminary insights into prophage-host interactions in agricultural environments. Overall, our results shed insight into the prophage repertoire of agriculturally-sourced strains of *Salmonella* and VTEC, which will provide important considerations when developing prophage-based control strategies.

## Data Availability Statement

The data presented in the study are deposited in the GenBank repository. The names of the repository and accession number(s) can be found below: https://www.ncbi.nlm.nih.gov/genbank/, BioProject IDs PRJNA6649825, PRJNA287560, and PRJNA649237.

## Author Contributions

KF and SW were responsible for study conception. SW provided funding for this research. KF and YL analyzed the data. TB and JF provided the bacterial strains used in this study. KF wrote the first draft of the manuscript and all authors provided feedback. All authors read and approved the final manuscript.

## Funding

This work was supported by the National Sciences and Engineering Research Council of Canada, NSERC Discovery Grant RGPIN-2015-04871. Funding for this project has also been provided by the Governments of Canada and British Columbia through the Canadian Agricultural Partnership, a federal-provincial-territorial initiative. The program is delivered by the Investment Agriculture Foundation of BC (INV 133).

## Author Disclaimer

Opinions expressed in this document are those of the author and not necessarily those of the Governments of Canada and British Columbia or the Investment Agriculture Foundation of BC. The Governments of Canada and British Columbia, and the Investment Agriculture Foundation of BC, and their directors, agents, employees, or contractors will not be liable for any claims, damages, or losses of any kind whatsoever arising out of the use of, or reliance upon, this information.

## Conflict of Interest

The authors declare that the research was conducted in the absence of any commercial or financial relationships that could be construed as a potential conflict of interest.

## Publisher's Note

All claims expressed in this article are solely those of the authors and do not necessarily represent those of their affiliated organizations, or those of the publisher, the editors and the reviewers. Any product that may be evaluated in this article, or claim that may be made by its manufacturer, is not guaranteed or endorsed by the publisher.
